# Clinically Proven Cardiac Sarcoidosis Presenting as Recurrent Ventricular Tachycardia With Severely Reduced Left Ventricular Function: A Case Report

**DOI:** 10.7759/cureus.109342

**Published:** 2026-05-21

**Authors:** Yatri Patel, Rahul Patel

**Affiliations:** 1 Internal Medicine, New Civil Hospital, Surat, IND; 2 Orthopedics and Traumatology, New Civil Hospital, Surat, IND

**Keywords:** angiotensin-converting enzyme, cardiac mri, cardiac sarcoidosis, corticosteroids, icd implantation, infiltrative cardiomyopathy, late gadolinium enhancement, left ventricular dysfunction, ventricular tachycardia

## Abstract

Cardiac sarcoidosis is a rare but life-threatening manifestation of systemic sarcoidosis, frequently presenting with ventricular arrhythmias, heart block, or sudden cardiac death. It remains underdiagnosed owing to its diverse clinical presentation and rarity and the limitations of conventional diagnostic modalities.

We report a case of a 42-year-old man with no prior cardiac history who presented with a three-day history of recurrent palpitations and three to four syncopal episodes. Electrocardiography during syncope demonstrated self-terminating ventricular tachycardia (VT), with one episode lasting five hours, requiring pharmacological cardioversion. Coronary angiography revealed entirely normal coronary arteries. Cardiac magnetic resonance imaging (CMR) with late gadolinium enhancement (LGE) identified a non-ischemic, epicardial-to-mid-myocardial enhancement pattern involving the interventricular septum, inferior wall, and lateral wall, accompanied by mediastinal and hilar lymphadenopathy. Two-dimensional echocardiography revealed severe left ventricular (LV) dysfunction with an ejection fraction of 15%. Serum angiotensin-converting enzyme levels were markedly elevated at 90 U/L (reference: 12-66 U/L). The patient was commenced on systemic corticosteroids (prednisolone), antiarrhythmic therapy (amiodarone), anticoagulation, and guideline-directed heart failure therapy.

This case underscores the diagnostic utility of CMR-LGE in identifying non-ischemic infiltrative cardiomyopathy and highlights the importance of considering cardiac sarcoidosis in patients presenting with unexplained VT and severely reduced LV function. Early immunosuppressive therapy and multidisciplinary management are pivotal in improving clinical outcomes.

## Introduction

Sarcoidosis is a multisystem granulomatous disorder of unknown aetiology, predominantly affecting the lungs and lymph nodes. Cardiac involvement, although clinically apparent in only 5% of cases, is identified at autopsy in up to 25%-30% of patients with systemic sarcoidosis [1]. Cardiac sarcoidosis can manifest as ventricular arrhythmias, high-degree atrioventricular block, dilated cardiomyopathy, or sudden cardiac death, making it one of the most lethal expressions of the disease [2].

The diagnosis of cardiac sarcoidosis is particularly challenging owing to the patchy and unpredictable distribution of granulomatous infiltration and the limited sensitivity of endomyocardial biopsy. Advances in cardiac magnetic resonance imaging (CMR) with late gadolinium enhancement (LGE) have transformed the diagnostic landscape, enabling non-invasive characterisation of myocardial fibrosis and inflammation [3]. Elevated serum angiotensin-converting enzyme (ACE) levels, mediastinal lymphadenopathy, and a non-ischemic LGE pattern collectively support the diagnosis in the appropriate clinical context. We report this case to emphasize the importance of considering cardiac sarcoidosis in patients with unexplained ventricular arrhythmias despite the lack of extracardiac disease.

The Heart Rhythm Society (HRS) 2014 diagnostic criteria for cardiac sarcoidosis outline two distinct diagnostic pathways [1]. The first pathway establishes a histological diagnosis, which is considered definite cardiac sarcoidosis, and requires myocardial tissue biopsy demonstrating non-caseating granulomas in the absence of any alternative identifiable cause. Although this approach offers high specificity, its sensitivity is limited because myocardial involvement in sarcoidosis is often patchy and may be missed on endomyocardial sampling.

The second pathway permits a clinical diagnosis, which is regarded as probable cardiac sarcoidosis, and requires histological confirmation of extracardiac sarcoidosis along with the presence of at least one of several cardiac features, provided other potential causes have been excluded. These cardiac features include a cardiomyopathy or heart block that responds to steroid or immunosuppressant therapy, an otherwise unexplained reduction in left ventricular ejection fraction (LVEF) below 40%, and unexplained sustained ventricular tachycardia, whether spontaneous or induced. The criteria are also satisfied by the presence of Mobitz type II second-degree atrioventricular block or complete (third-degree) heart block, patchy myocardial uptake on cardiac fluorodeoxyglucose positron emission tomography (FDG-PET) in a pattern consistent with cardiac sarcoidosis, late gadolinium enhancement on cardiac magnetic resonance imaging in a distribution consistent with cardiac sarcoidosis, or positive gallium uptake within the myocardium.

Herein, we present a case of a 42-year-old male patient with no prior medical history who developed recurrent ventricular tachycardia and syncopal episodes, and was ultimately diagnosed with cardiac sarcoidosis based on CMR findings, elevated ACE levels, which supported the diagnosis further, and mediastinal lymphadenopathy.

## Case presentation

A 42-year-old male patient (identity anonymized per institutional policy) presented to the emergency department with a three-day history of recurrent palpitations and, on the third day, three to four witnessed syncopal episodes. Each palpitation episode lasted approximately 15-20 minutes, with the last palpitation episode associated with sustained ventricular tachycardia lasting five hours, requiring pharmacological cardioversion on admission. There was no preceding prodrome, chest pain, or breathlessness before the syncopal events. The patient denied any personal or family history of cardiac disease and had no known medical comorbidities, prior surgical interventions, regular medications, or drug allergies.

On presentation, the patient was haemodynamically stable, with blood pressure 110/66 mmHg, heart rate during syncopal attack 150 beats/minute, and oxygen saturation 99% on room air. General examination was unremarkable with no peripheral lymphadenopathy or skin lesions, and no respiratory abnormality. The ECG showed sustained ventricular tachycardia (Figure 1).

**Figure 1:**
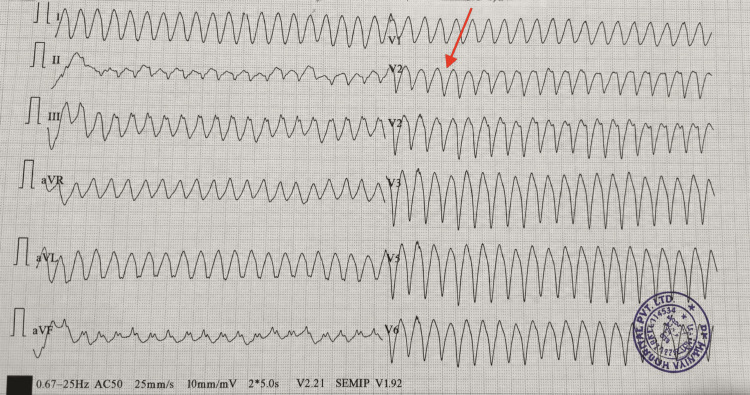
ECG at the time of presentation Rate: approximately 150–180 bpm (very rapid, regular complexes); rhythm: regular, fast, wide-complex throughout most leads; QRS morphology: wide, bizarre-looking complexes visible across all 12 leads—the hallmark of ventricular origin. The arrow points to the area in leads V1/V2 where there is a visible transition—the onset of the VT run. Concordance: precordial leads (V1–V6) appear to show predominantly similar deflection directions, consistent with ventricular tachycardia. No clear P waves were discernible before QRS complexes, supporting a ventricular origin.

Coronary angiography (CAG) demonstrated normal coronary anatomy with thrombolysis in myocardial infarction (TIMI) grade 3 flow in all major epicardial vessels and no obstructive or flow-limiting disease, effectively excluding an ischemic aetiology for the observed wall motion abnormalities and arrhythmia, as shown in Figure 2.

**Figure 2:**
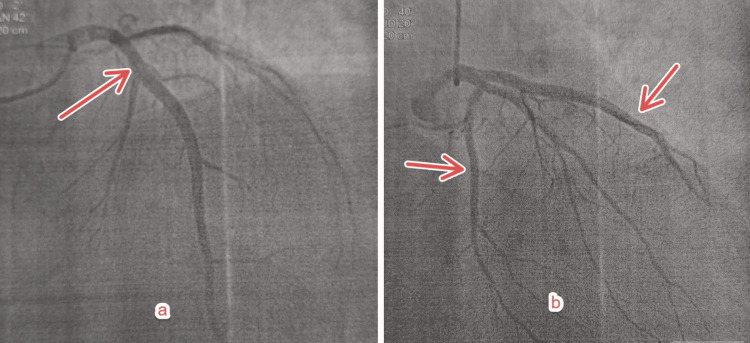
Coronary angiography (a) The image shows the left main (LM) coronary artery bifurcating into the left anterior descending artery (LAD), and running downward, the left circumflex (LCx) artery. The arrow points to the LAD and its diagonal branches. Vessel walls appear smooth, with no filling defects, stenosis, or cutoffs. (b) The horizontal arrow points to the mid-LAD. The diagonal arrow points to a diagonal branch (D1 or D2); vessels show normal, smooth opacification.

Two-dimensional (2D) transthoracic echocardiography revealed severe left ventricular systolic dysfunction (Figure 3). Regional wall motion abnormalities (RWMA) were identified across the interventricular septum, lateral wall, and inferior wall, with severe hypokinesia throughout. Mild pericardiac effusion was also noted, as shown in Figure 3. The right ventricle was mildly dilated, and pulmonary artery systolic pressure (PASP) was estimated at right atrial pressure (RAP) + 29 mmHg. Inferior vena cava (IVC) congestion was noted, consistent with elevated RAP as explained in Table 1.

**Figure 3:**
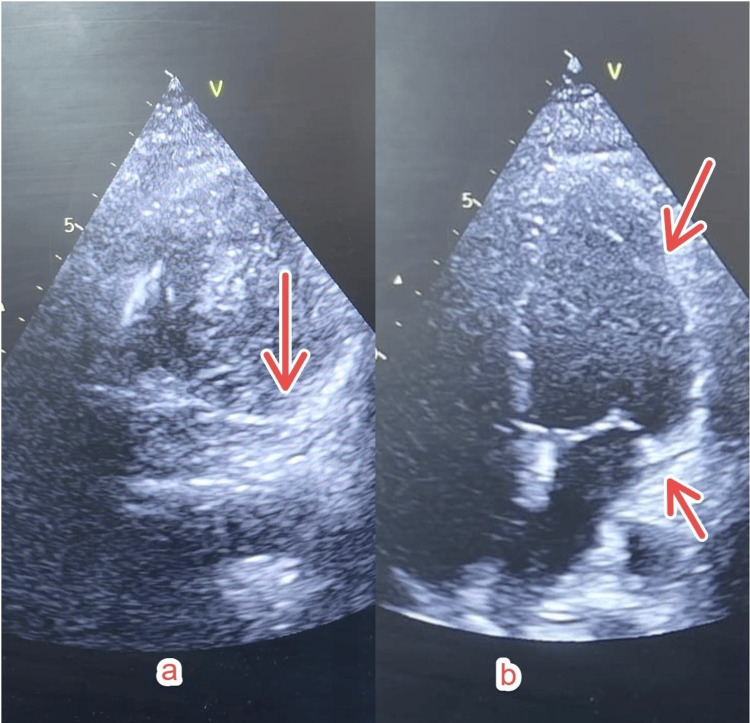
Transthoracic echocardiography (a) A standard cardiac view (parasternal long axis): the arrow points to what appears to be a large echo-free (black/dark) space within or around the cardiac chambers; this dark space represents pericardial effusion. (b) The upper arrow points to a bright echogenic structure near the anterior wall/pericardium—thickened pericardium, fibrinous deposits. The lower arrow points to what appears to be valve structures or internal echoes within the effusion/chamber—mitral or tricuspid valve apparatus, fibrin strands within the effusion.

**Table 1:** Transthoracic echocardiography The transthoracic echocardiography findings demonstrated significant structural and functional cardiac abnormalities. Regional wall motion abnormalities were present, involving the entire IVS, lateral wall, and the inferior wall regions. The left ventricle was mildly dilated with severe systolic dysfunction, reflected by a markedly reduced ejection fraction of 15%. The left atrium was also mildly dilated. Mild mitral and tricuspid regurgitation was also noted, and the inferior vena cava appeared congested. RWMA: regional wall motion abnormality; IVS: interventricular septum; PASP: pulmonary artery systolic pressure; IVC: inferior vena cava; LVEF: left ventricular ejection fraction; RAP: right atrial pressure

Echocardiographic parameter	Finding
RWMA	Positive—whole IVS, lateral wall, inferior wall
Degree of hypokinesia	Severe—IVS, lateral wall, inferior wall
Left atrium	Mildly dilated (36 mm)
Left ventricle	Mildly dilated with severe systolic dysfunction (48 mm)
Mitral regurgitation	Mild
Tricuspid regurgitation	Mild
PASP	RAP + 29 mmHg
IVC	Congested—elevated RAP (19 mm)
LVEF	15%—severely reduced

Cardiac magnetic resonance imaging with late gadolinium enhancement demonstrated a non-ischemic distribution of myocardial fibrosis, with patchy, multifocal enhancement (Figure 4). Specific findings included non-ischemic epicardial-to-mid-myocardial enhancement in the basal-to-mid anteroseptal and inferoseptal segments, which suggested cardiac sarcoidosis. Enhancement involving the basal inferior wall, distal septum, and inferior wall, and also focal epicardial enhancement could be seen. There were multiple enlarged hilar lymph nodes (left-sided predominance), with subcarinal and carinal lymphadenopathy seen.

**Figure 4:**
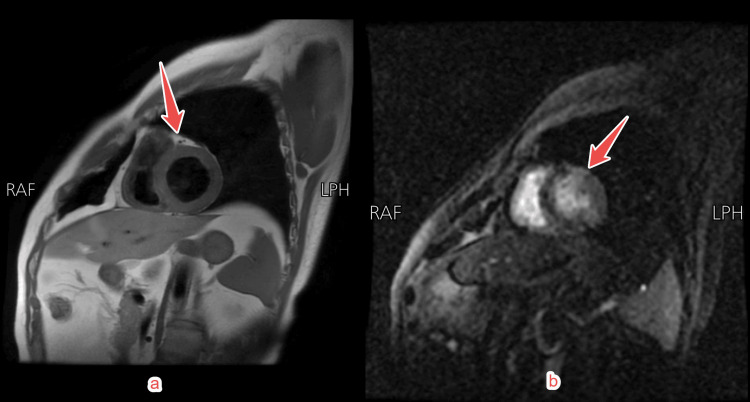
Cardiac magnetic resonance imaging (a) Anatomical/morphologic sequence: This is the T1- or cine-type image showing structural detail—the cross-sectional view of the heart, with the ventricular walls and chambers, surrounding lung, and the chest wall. The arrow points to a segment of the myocardium—in cardiac sarcoidosis, the wall here shows abnormal thickening during the active inflammatory phase. The patchy, regional nature of the wall change (rather than uniform involvement) is a clue to an infiltrative/granulomatous process rather than ischemic disease. (b) Late gadolinium enhancement (LGE) sequence: the arrow points to the bright focus in the myocardium. That bright signal represents myocardial edema—increased tissue water from active granulomatous inflammation. This is one of the hallmark MRI findings of active cardiac sarcoidosis, and matching it to the same location in panel (a) shows that the structurally abnormal segment is also inflamed and active.

These findings were consistent with an infiltrative cardiomyopathy of granulomatous or sarcoid aetiology. Correlation with serum ACE was recommended for diagnostic confirmation per established criteria [4,5]. Haematological and biochemical parameters are presented in Table 2, which show increased ACE levels suggesting sarcoidosis, and, along with imaging findings, highly support the diagnosis of cardiac sarcoidosis.

**Table 2:** Hematological and biochemical investigation Complete blood count and serum electrolytes were within reference ranges, and renal function was preserved. Cardiac biomarker troponin I was within normal limits, suggesting no ongoing myocardial injury. The angiotensin-converting enzyme (ACE) level was significantly elevated (90 U/L), which serves as supportive biochemical evidence for sarcoidosis in the appropriate clinical context. Screening for infectious etiologies, including hepatitis B, HIV, and hepatitis C, was negative.

Investigation	Result	Reference range
Hemoglobin	13.4 g/dL	12.0–16.0 g/dL
WBC count	6,800/µL	4,000–11,000/µL
Platelet count	256,000/µL	150,000–400,000/µL
Sodium (Na⁺)	138 mEq/L	136–145 mEq/L
Potassium (K⁺)	4.1 mEq/L	3.5–5.0 mEq/L
Chloride (Cl⁻)	102 mEq/L	98–107 mEq/L
Magnesium (Mg²⁺)	2.1 mEq/L	1.7–2.4 mEq/L
Creatinine	0.8 mg/dL	0.7–1.3 mg/dL
Troponin I	0.2 ng/mL	0.04–0.4 ng/mL
ACE level	90 U/L ↑	12–66 U/L
HBsAg/HIV/HCV	Negative	Negative

The patient was started on early corticosteroid therapy, receiving an initial stat dose of 500 mg followed by a maintenance regimen of 50 mg orally once daily. Guideline-directed heart failure management was initiated concurrently, which included oral amiodarone at a dose of 200 mg once daily along with metoprolol 50 mg once daily. In addition, a single-chamber automated implantable cardioverter-defibrillator (AICD) was implanted, with intraoperative parameters demonstrating a pacing threshold of 0.9 V, a ventricular lead impedance of 853 ohms, and a programmed rate response range of 40 to 130 beats per minute. On further follow-up, the patient showed symptomatic improvement, as there was no episode of syncope or palpitation; also, the LVEF improved from 15% to 25%.

## Discussion

This case illustrates several important clinical and diagnostic facets of probable cardiac sarcoidosis. The patient was entirely asymptomatic from a cardiac standpoint before this presentation, consistent with the insidious and clinically silent nature of myocardial granulomatous infiltration. Ventricular tachycardia was the sentinel manifestation - a pattern well described in the cardiac sarcoidosis literature, where arrhythmia frequently precedes overt heart failure [1,2].

The diagnostic algorithm in this case was methodical and evidence-based. Coronary angiography that demonstrated TIMI 3 flow unequivocally excluded obstructive coronary artery disease. Cardiac magnetic resonance then demonstrated a non-ischemic, epicardial-predominant enhancement pattern - a hallmark of cardiac sarcoidosis that contrasts with the subendocardial or transmural distribution typical of ischemic cardiomyopathy [3]. The predilection for the basal interventricular septum and inferior wall in this patient is consistent with established cardiac magnetic resonance imaging patterns in cardiac sarcoidosis [6].

A markedly elevated ACE level of 90 U/L provided crucial corroborating evidence. Although ACE lacks sensitivity and specificity when used in isolation, its elevation in the context of characteristic cardiac magnetic resonance imaging findings and mediastinal lymphadenopathy substantially increases the diagnostic probability for sarcoidosis [4]. The presence of bilateral hilar and subcarinal lymphadenopathy further supports systemic sarcoid involvement.

The severely reduced left ventricular ejection fraction of 15% observed at presentation highlights the extent of myocardial compromise. Such profound left ventricular dysfunction in cardiac sarcoidosis is associated with adverse outcomes, including progressive heart failure and sudden cardiac death [2,5]. Regarding device therapy, current HRS guidelines recommend ICD implantation in patients with cardiac sarcoidosis and sustained ventricular tachycardia or left ventricular ejection fraction ≤35%, irrespective of response to immunosuppression [7].

Corticosteroid therapy remains the cornerstone of immunosuppressive management in cardiac sarcoidosis, with evidence suggesting improvement in LVEF and reduction in arrhythmia burden following treatment [8]. Histological confirmation remains the gold standard for diagnosis; however, due to the patchy nature of myocardial involvement, endomyocardial biopsy has limited sensitivity. Therefore, extracardiac tissue sampling, such as mediastinal lymph node biopsy, is often preferred when feasible [9].

Cardiac magnetic resonance imaging has emerged as a highly sensitive modality for detecting myocardial involvement in sarcoidosis, with studies demonstrating its utility in identifying non-ischemic patterns of fibrosis [10]. Adjunctive imaging with FDG-PET can further aid in identifying active myocardial inflammation and guiding therapy [11]. Systematic reviews have also supported the role of corticosteroids in improving clinical outcomes in cardiac sarcoidosis [12].

The differential diagnosis in this case includes giant cell myocarditis, arrhythmogenic cardiomyopathy, and other infiltrative cardiomyopathies. Giant cell myocarditis may present with ventricular tachycardia and conduction abnormalities similar to cardiac sarcoidosis, but often demonstrates a more fulminant clinical course and may require more aggressive immunosuppressive therapy. Arrhythmogenic cardiomyopathy was considered less likely given the imaging findings and absence of characteristic fibrofatty myocardial replacement. Other infiltrative disorders, such as amyloidosis, were also considered but were not supported by the clinical and imaging profile.

## Conclusions

This case demonstrates that clinically suspected cardiac sarcoidosis should be considered in any patient presenting with unexplained ventricular tachycardia and non-ischemic cardiomyopathy, even in the absence of prior systemic disease. The integration of cardiac magnetic resonance imaging, serum ACE quantification, and coronary angiography provides a robust non-invasive diagnostic framework. Histological confirmation via accessible tissue biopsy and multimodality imaging follow-up should be pursued where feasible or if the diagnosis is not evident. Corticosteroid therapy remains the cornerstone of immunosuppressive management. In this case, the diagnosis was clinically proven, and the patient responded well to the treatment, hence avoiding the invasive procedures.
